# RETRACTION: Nesfatin-1/Nucleobindin-2 Is a Potent Prognostic Marker and Enhances Cell Proliferation, Migration, and Invasion in Bladder Cancer

**DOI:** 10.1155/dim/9803765

**Published:** 2025-04-26

**Authors:** Disease Markers

RETRACTION: G.-M. Liu, Z.-Q. Xu, and H.-S. Ma. “Nesfatin-1/Nucleobindin-2 Is a Potent Prognostic Marker and Enhances Cell Proliferation, Migration, and Invasion in Bladder Cancer,” *Disease Markers*, no. 2018 (2018): 1–9. https://doi.org/10.1155/2018/4272064.

The above article, published online on 19 September 2018 in Wiley Online Library (wileyonlinelibrary.com), has been retracted by John Wiley & Sons Ltd.

The retraction has been agreed following an investigation of the concerns raised by *Hoya camphorifolia* on PubPeer [1], which identified multiple instances of inappropriately overlapping figures. Our investigation concluded that there are concerns about the reliability of this article.

More specifically:

- Figure 3a: The mirror image of the 4 cell plates appears in 3 publications, where the cultures are labelled as a different cell line. These include:

• Figure 4a of [2]
• Figure 4a of [3]
• Figure 3a of [4]

- Figure 4a: The “T24 Cell - Control” and“ 5637 cell - Control” panels are identical to the bottom 2 panels of Figure 4a from [5], where they are described as “T24 Cell - shRNA” and“ 5637 cell - shRNA”, respectively. The images also appear as “T24 Cell - Control” and“ 5637 cell - Control” in Figure 4a of [6].

- Figure 4b: The “5638 cell- shRNA” image (bottom right) appears in the bottom right of Figure 3d of [7] and Figure 3d of [8]. The other 3 panels of the figure have also been duplicated in Figure 4b of [6].

- Figure 5a: The third and fourth tumors in the top row and the second tumor of the bottom row appear identical to the second and third tumors in top row and the first tumor in the bottom row of Figure 4a of [9].

The authors were informed of the decision to retract the article but did not provide a response.
